# Exploring the Structural Characteristics and Antioxidant Capacity of Pectins from *Adenophora tetraphylla* (Thunb.) Fisch.

**DOI:** 10.3390/molecules30061301

**Published:** 2025-03-13

**Authors:** Su Yan, Shuo Zhang, Yuxuan Liu, Hao Zang, Lihui Zhang, Duo Liu

**Affiliations:** 1 School of Life Sciences, Changchun Normal University, Changchun 130032, China; yansu@ccsfu.edu.cn (S.Y.); zhangshuo727720@163.com (S.Z.); 13044055841@163.com (Y.L.); 2 School of Pharmacy and Medicine, Tonghua Normal University, Tonghua 134002, China; zanghao@thnu.edu.cn

**Keywords:** *Adenophora tetraphylla* (Thunb.) Fisch., pectic polysaccharides, structural domain, structural analysis, antioxidant ability

## Abstract

This research explores the structural composition and antioxidant abilities of pectins extracted from *Adenophora tetraphylla* (Thunb.) Fisch. Pectins, which are a complex group of acidic polysaccharides, exhibit various biological activities due to their unique structural domains. Following aqueous extraction, the pectins underwent sequential purification using ion exchange and gel permeation chromatography techniques. FT-IR and NMR techniques were used to elucidate their structural characteristics. The structural investigation was enhanced through the application of multiple characterization methods: Congo red binding analysis, circular dichroism measurements, and scanning electron microscopy imaging. Among the isolated pectins, WATP-A2b (22.5 kDa) and WATP-A3b (49.8 kDa) demonstrated significant variations in their structural domain organization, comprising different ratios of homogalacturonan, rhamnogalacturonan I, and rhamnogalacturonan II. WATP-A3b displayed the most potent antioxidant performance among the tested pectins, effectively scavenging all three free radical species, which may be correlated with its higher galacturonic acid proportion and substantial rhamnogalacturonan I domain presence. These experimental results provide valuable insights into the correlation between structural characteristics and biological functions of pectins derived from *Adenophora tetraphylla* and their potential applications in healthcare.

## 1. Introduction

As the outermost layer of plant cells, the cell wall plays crucial roles in environmental interactions, growth and development regulation, and intercellular communication. This structurally complex matrix consists of diverse polysaccharides (including cellulose and pectin), enzymes, and structural proteins, whose composition dynamically changes during plant growth and environmental adaptation. Pectin, a major component of primary plant cell walls, is initially secreted into the cell wall in a highly methyl-esterified form and subsequently undergoes de-esterification mediated by pectin methylesterases within the cell wall. The degree of pectin methyl esterification, particularly in the homogalacturonan (HG) domains, undergoes significant modifications throughout plant growth and development [1,2]. Pectins, which constitute a class of acidic polysaccharides in plant cellular structures, have been widely recognized for their diverse bioactivities encompassing antioxidant potential, anti-inflammatory responses, and glucose-lowering effects [3]. The architectural organization of pectin polysaccharides features three primary structural domains: HG, rhamnogalacturonan I (RG-I), and rhamnogalacturonan II (RG-II). Dominating the pectin composition at roughly 65%, the HG domain forms a straight-chain polymer of *α*-(1→4)-linked galacturonic acid (GalA) residues, which are often chemically altered through methylation at the *O*-3 position and/or acetylation at either *O*-2 or *O*-3 positions [4]. The RG-I domain, representing approximately one-fifth to one-third of pectin’s composition, is characterized by a unique repeating pattern of [→2)-*α*-Rha*p*-(1→4)-*α*-GalA*p*-(1→] units. These structural units serve as attachment points for diverse side chains at the C4 position of the (1→2)-*α*-Rha*p* units, and are side chains that include arabinan, galactan, arabinogalactan-I, and arabinogalactan-II (AG-II). The proportion of neutral sugar side chains in RG-I varies widely, ranging from 25% to 80%, depending significantly on the source of the pectin and the extraction methods employed. Additionally, as a fundamental constituent of plant cellular architecture, the RG-II domain demonstrates exceptional evolutionary preservation in its molecular sequence, demonstrating a remarkable level of uniformity across different plant species [5]. This conservation suggests its essential role in the pectin structure and function, potentially influencing cell wall integrity and plant growth.

With their substantial GalA composition enabling superior radical neutralization capabilities, pectins have emerged as valuable antioxidant candidates. The combination of their unique molecular properties and favorable safety profile has attracted considerable research attention [6]. Pectins, with their macromolecular structure, scavenge free radicals and mitigate reactive oxygen species through metal ion chelation and peroxide scavenging. Although phenolic compounds may react more quickly in vitro due to their lower molecular weight, pectins, with their high molecular weight and multifunctionality, offer broader and more sustained antioxidant protection in vivo. The abundance of pectins in plant cell walls, as well as their safe consumption history and biocompatibility, ensures their favorable safety profile [7]. As sophisticated biopolymers, pectins demonstrate variable radical scavenging activities that are dependent on their physicochemical properties, encompassing solubility parameters, molecular dimensions, and architectural details including monosaccharide profiles, glycosidic linkages, and three-dimensional configurations [8]. Pectin molecules demonstrate both conserved structural features and source-specific variations across different plant species [9]. Their bioactivity is profoundly influenced by domain-specific compositional and structural differences. Although the fundamental chemical architecture of pectins is well characterized, species-dependent variations in side-chain configuration, esterification patterns, molecular dimensions, and branching complexity contribute to functional diversity [10]. Consequently, comprehensive structural elucidation of diverse pectins is crucial for establishing structure–function correlations and optimizing their therapeutic potential in pharmaceutical applications.

In recent scientific developments, pectin research focusing on monocotyledonous sources has gained significant momentum, becoming a prominent field of study [11,12,13]. Within this expanding domain, *Adenophora potaninii*, a member of the *Adenophora* genus, has attracted substantial research focus, resulting in the successful isolation and characterization of two distinct polysaccharide molecules from this plant species [14].

The medicinal material known as *Adenophorae radix*, commonly referred to as Nan Shashen in Chinese medicine practice, is obtained from the dried roots of either *Adenophora tetraphylla* (Thunb.) Fisch. (*Adenophora tetraphylla*) or *Adenophora stricta* Miq., both from the genus *Adenophora* in the Campanulaceae family. This medicinal root is known for its numerous health benefits, including immune regulation, heart protection, pain relief, and cough suppression [15]. This medicinal herb contains a spectrum of phytochemicals, notably cycloartenyl acetate, *β*-sitosterol, taraxerone, lupenone, octacosanoic acid, and praeruptorin A [16].

Polysaccharides are crucial active ingredients in *Adenophorae radix*, comprising 5–14% of its dry weight. Employing response surface methodology, Zhang’s research team optimized the polysaccharide extraction process, determining that a temperature of 72.5 °C, duration of 133 min, and material-to-solvent ratio of 1:35 yielded maximum polysaccharide recovery at 5.68%. They also identified eight monosaccharides in *Adenophorae radix* polysaccharides, with Ara, Gal, Glc, and GalA being predominant [17]. Using modified extraction parameters, Li and co-workers operated at 90 °C for 180 min, with a material-to-solvent ratio of 1:10, achieving a crude polysaccharide yield of 13.9%. They further isolated RAPS (18.0 kDa) from *Adenophorae radix* using DEAE-52 and Sephacryl S-300HR columns [18].

The yield of *Adenophora potaninii* polysaccharides can vary significantly depending on the extraction conditions. These polysaccharides include both neutral and acidic types. Chen et al., employing DEAE cellulose and Sepharose G-200, obtained two polysaccharides with molecular weights of 83.0 kDa (AP-1) and 63.0 kDa (AP-3), respectively. AP-1 is identified as dextran, while AP-3 is an acidic heteropolysaccharide consisting of Glc, Rha, Ara, and GlcA in a 5:1:1:3 ratio [14]. Cutting-edge pharmacological research has uncovered that *Adenophora potaninii* polysaccharides demonstrate a spectrum of biological activities, encompassing antioxidant capacity, aging prevention, neurocognitive restoration, radioprotective effects, immunostimulation, and liver protection [19,20].

Despite their potential significance, the structural characteristics and corresponding bioactivities of *Adenophora tetraphylla* pectins have received limited scientific attention. The current research seeks to address this knowledge gap by developing an extraction protocol for these pectins, determining their molecular structure through comprehensive analytical approaches, and investigating both their free radical scavenging capabilities and the underlying structure–activity relationships.

## 2. Results and Discussion

### 2.1. Extraction of Pectins from Adenophora tetraphylla

Water-soluble *Adenophora tetraphylla* polysaccharide (WATP) was acquired from *Adenophora tetraphylla* via thermal aqueous extraction with subsequent ethanol precipitation, achieving a yield of 5.4% based on the dry weight (Table 1). The contents of total carbohydrate, glucuronic acid, and protein in WATP were found to be 61.7%, 26.5%, and 2.0%, respectively. Monosaccharide analysis revealed that WATP comprised GalA, Rha, Gal, Ara, Glc, GlcA, Xyl, and Man in molar ratios of 25.4:6.5:16.2:23.2:19.3:3.6:1.2:3.6 (Table 1).

**Figure 1:**
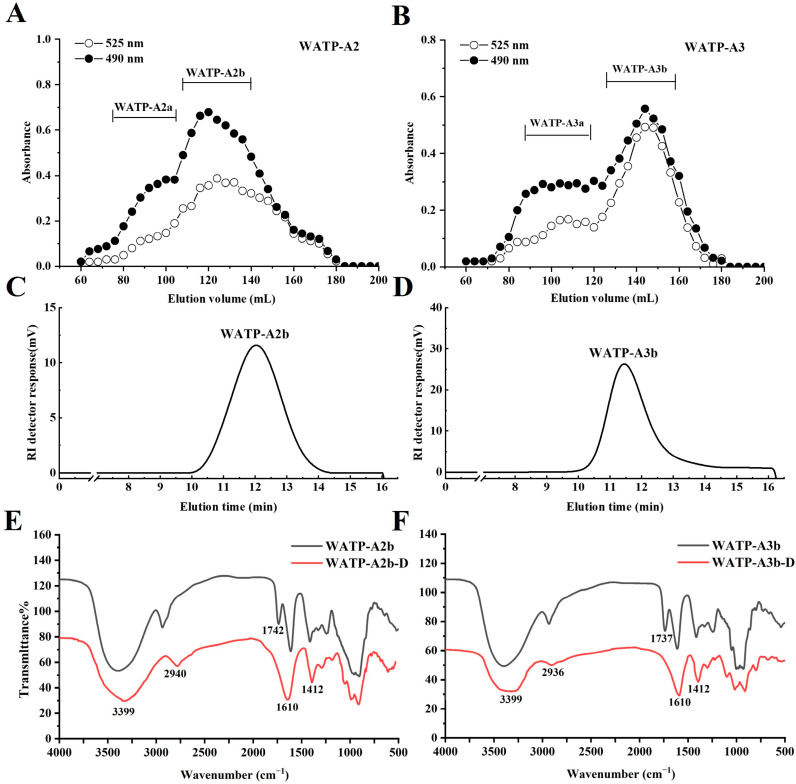
Structural and chemical properties of WATP-A2b and WATP-A3b fractions. (**A**,**B**) Elution profiles on Sepharose CL-6B column, (**C**,**D**) HPGPC profiles, and (**E**,**F**) FT-IR spectral data.

WATP was fractionated using ion-exchange chromatography, yielding WATP-N (43.1% of WATP) and WATP-A (23.5% of WATP). WATP-A was further separated by DEAE–cellulose chromatography into four subfractions: WATP-AH (10.6%), WATP-A2 (20.3%), WATP-A3 (42.0%), and WATP-A5 (8.1%). Further purification of WATP-A2 and WATP-A3 using Sepharose CL-6B column chromatography (Figure 1A,B) resulted in two major purified fractions: WATP-A2b (79.5%) and WATP-A3b (55.0%). Carbohydrate analysis of WATP-A2b and WATP-A3b showed nearly identical monosaccharide patterns, dominated by GalA, Rha, Gal, Ara, and Glc (constituting roughly 95% of the total monosaccharides), with small proportions of GlcA, Xyl, and Man (Table 1). The presence of GalA, Rha, Gal and Ara implied the structural incorporation of both HG and RG-I domains. Confirmation of RG-II domains was obtained through positive thiobarbituric acid (TBA) assay results for both fractions.

### 2.2. Evaluation of Purity, Molecular Homogeneity, and Weight Characteristics of WATP-A2b and WATP-A3b

**Figure 2:**
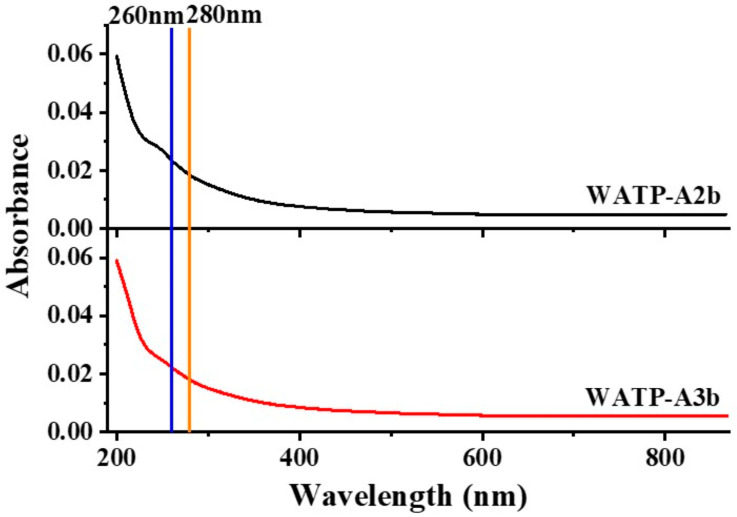
Ultraviolet–visible spectra of WATP-A2b and WATP-A3b.

The absence of UV absorption at 260 and 280 nm in both WATP-A2b and WATP-A3b (Figure 2) verified the effective elimination of proteinaceous and nucleic acid impurities, confirming the high purity of isolated pectins. High-performance gel permeation chromatography (HPGPC) analysis demonstrated fraction homogeneity through the observation of sharp, symmetrical elution profiles (Figure 1C,D). Molecular weight analysis established the polymer sizes as 22.5 kDa for WATP-A2b and 49.8 kDa for WATP-A3b.

### 2.3. Fourier-Transform Infrared (FT-IR) Spectroscopic Evaluation of WATP-A2b and WATP-A3b

FT-IR spectroscopy serves as a valuable analytical tool for elucidating the fundamental structural characteristics of polysaccharides, enabling the identification of functional group composition and carbohydrate residue configurations through spectral analysis [21]. As shown in Figure 1E,F, the pre-processed FT-IR spectra analysis revealed that the two fractions share remarkably similar spectral patterns, displaying typical pectin-specific absorption features. The intense 3399 cm^−1^ band represented O−H stretching vibrations, reflecting hydrogen bonding interactions in polysaccharide chains. Weaker absorptions near 2940 and 2936 cm^−1^ were assigned to asymmetric C−H stretching vibrations in aliphatic groups. Characteristic peaks at 1740, 1610, and 1245 cm^−1^ provided spectral evidence for uronic acid presence [22]. Distinct infrared absorptions at 1740 cm^−1^ and 1610 cm^−1^ were identified as characteristic vibrations of esterified and non-esterified carboxyl groups in GalA residues, respectively [23]. The presence of both *α*- and *β*-glycosidic bonds was confirmed by characteristic bands in the 825.2–831.2 cm^−1^ and 894.1–900.1 cm^−1^ regions. The degree of methylation (DM) was calculated using the formula DM (%) = [A1740/(A1740 + A1610)] × 100%, where A1740 and A1610 correspond to the peak areas of esterified and free carboxyl groups [24]. Quantitative analysis yielded DM values of 27.5% for WATP-A2b and 31.0% for WATP-A3b, confirming their classification as low-methyl-esterified pectins.

### 2.4. NMR Analysis of WATP-A2b and WATP-A3b

#### 2.4.1. NMR Analysis of WATP-A2b

**Figure 3:**
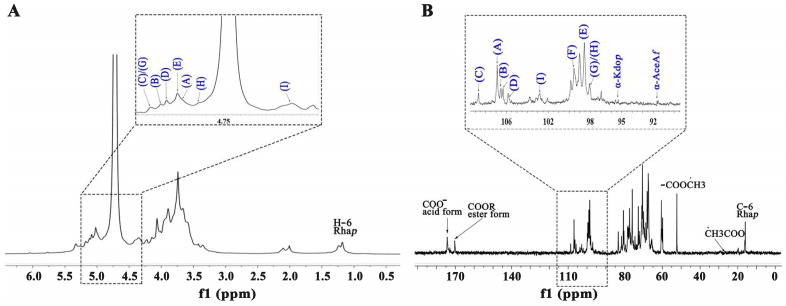
NMR spectral characterization of WATP-A2b: (**A**) ^1^H NMR spectrum; (**B**) ^13^C NMR spectrum (spectral labels A–I correspond to residue assignments detailed in Table 2).

**Figure 4:**
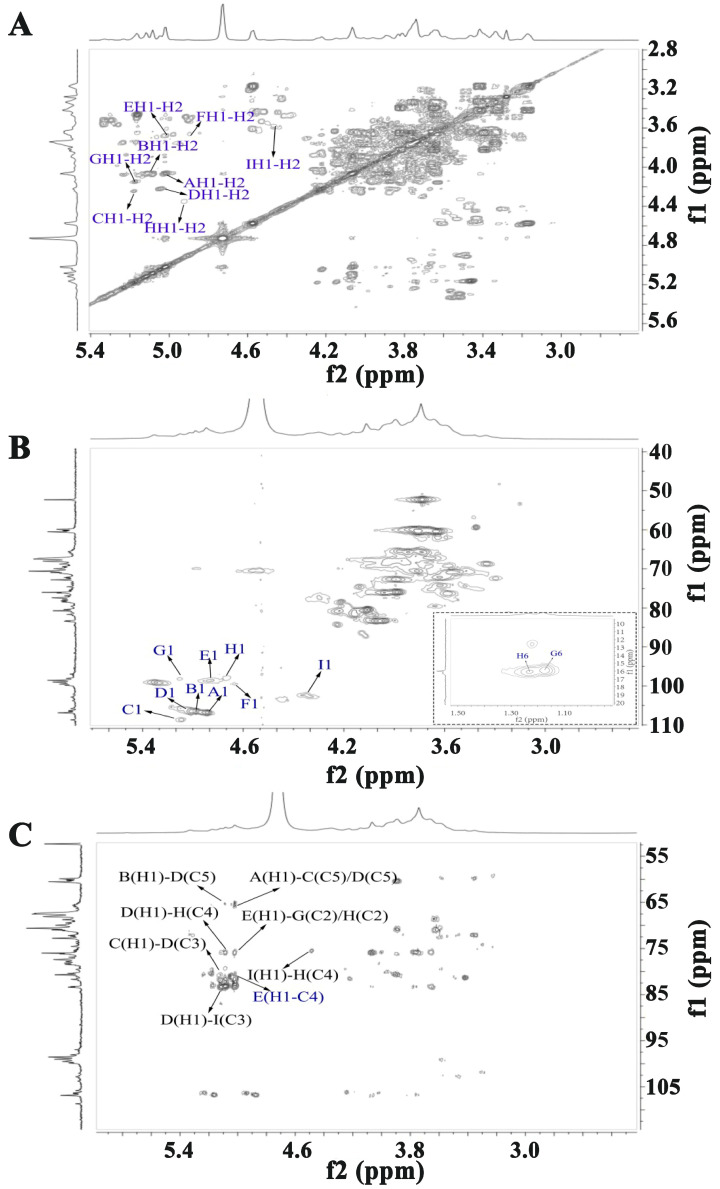
Structural elucidation of WATP-A2b by 2D NMR: (**A**) COSY spectrum; (**B**) HSQC spectrum; (**C**) HMBC spectrum (residue designations A–I correspond to chemical shift data in Table 2).

Figure 3 and Figure 4 present the one- and two-dimensional NMR spectra of WATP-A2b. Analysis of the ^1^H NMR spectrum (Figure 3A) identified anomeric proton signals in the 4.41–5.18 ppm range, demonstrating the presence of *α*- and *β*-configured carbohydrate units [25], with some spectral overlap observed. The ^13^C NMR spectrum (Figure 3B) exhibited anomeric carbon resonances spanning 91.77–108.62 ppm. Nine major ^1^H-^13^C coupling sugar-residue-related peaks were observed in the ^1^H-^13^C HSQC, with values corresponding to various residues labeled A-I. These results indicate that WATP-A2b mainly comprises *α*- and *β*-configuration sugar residues. The C2-C6 signals of the nine residues are distributed in the region of 60–83 ppm, suggesting that WATP-A2b contains pyran-type and furan-type sugar residues [25], consistent with FT-IR spectroscopic findings. The signal peak at 1.17–1.23 ppm corresponds to -CH_3_ of Rha. The strong signal at 3.72 ppm is attributed to -COOCH_3_ [26]. The signal at 15.98 ppm in the ^13^C NMR spectrum belongs to C-6 of Rha. The strong signal at 52.14 ppm corresponds to -COOCH_3_ methyl carbon. A distinct resonance at 19.58 ppm was assigned to the methyl carbon of acetyl groups. The presence of weaker signals at 91.77 and 95.86 ppm, corresponding to C2 atoms of *α*-kdop and *α*-Ace*f*A, indicated minor RG-II domain content in WATP-A2b, consistent with TBA findings. Utilizing combined 2D NMR techniques (^1^H-^1^H COSY, Figure 4A; ^1^H-^13^C HSQC, Figure 4B; HMBC, Figure 4C), the complete proton and carbon chemical environments of all carbohydrate residues were elucidated and are tabulated in Table 2.

HMBC spectral analysis of WATP-A2b revealed its complex structural organization. HMBC spectral analysis revealed key structural features through specific correlations: the 5.01/81.61 ppm (EH1-EC4) cross-peak identified [→4)-GalA*p*-(1→4)-GalA*p*-(1→] units, while the 5.01/75.91 ppm (EH1-GC2/HC2) signal indicated the coexistence of [(→4)-*α*-GalA*p*-(1→2)-*α*-Rha*p*-(1→] and [→4)-*α*-GalA*p*-(1→2,4)-*α*-Rha*p*-(1→] sequences. Structural analysis reveals that WATP-A2b contains HG and RG-I domains, supported by HMBC correlations at 5.08/76.06 ppm (DH1-HC4) and 4.41/76.06 ppm (IH1-HC4), which indicate (1→3,5)-*α*-Ara*f* and (1→3)-*β*-Gal*p* connections to C4 of (1→2,4)-*α*-Rha*p*. HMBC spectral analysis revealed critical correlations at 5.16/80.45 ppm (CH1-DC3), 5.02/65.80 ppm (AHI-CC5), and 5.11/65.76 ppm (BH1-DC5), indicating that *α*-Ara*f* residues exclusively occupy side-chain positions. The arabinan side chains, formed by t-*α*-Ara*f*, (1→5)-*α*-Ara*f*, and (1→3,5)-*α*-Ara*f*, are attached to the RG-I backbone via (1→3,5)-*α*-Ara*f* linkages. The key HMBC cross-peak at 5.08/76.06 ppm (DH1-IC3) illustrates AG-II domain formation through (1→3,5)-*α*-Ara*f* attachment to C3 of (1→3)-*β*-Gal*p*, which links to RG-I. Terminal *α*-Ara*f* units are positioned at C5 of both (1→5)-*α*-Ara*f* and (1→3,5)-*α*-Ara*f*. These structural features demonstrate that WATP-A2b’s backbone primarily contains HG, RG-I, and RG-II domains, with AG-II and arabinan side chains connected to RG-I at C4 of (1→2,4)-*α*-Rha*p* via (1→3,5)-*α*-Ara*f* and (1→3)-*β*-Gal*p* residues.

#### 2.4.2. NMR Analysis of WATP-A3b

**Figure 5:**
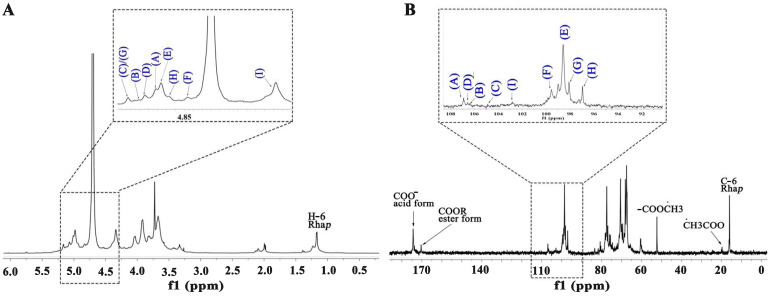
NMR spectral characterization of WATP-A3b: (**A**) ^1^H NMR spectrum; (**B**) ^13^C NMR spectrum (spectral labels A–I correspond to residue assignments detailed in Table 3).

**Figure 6:**
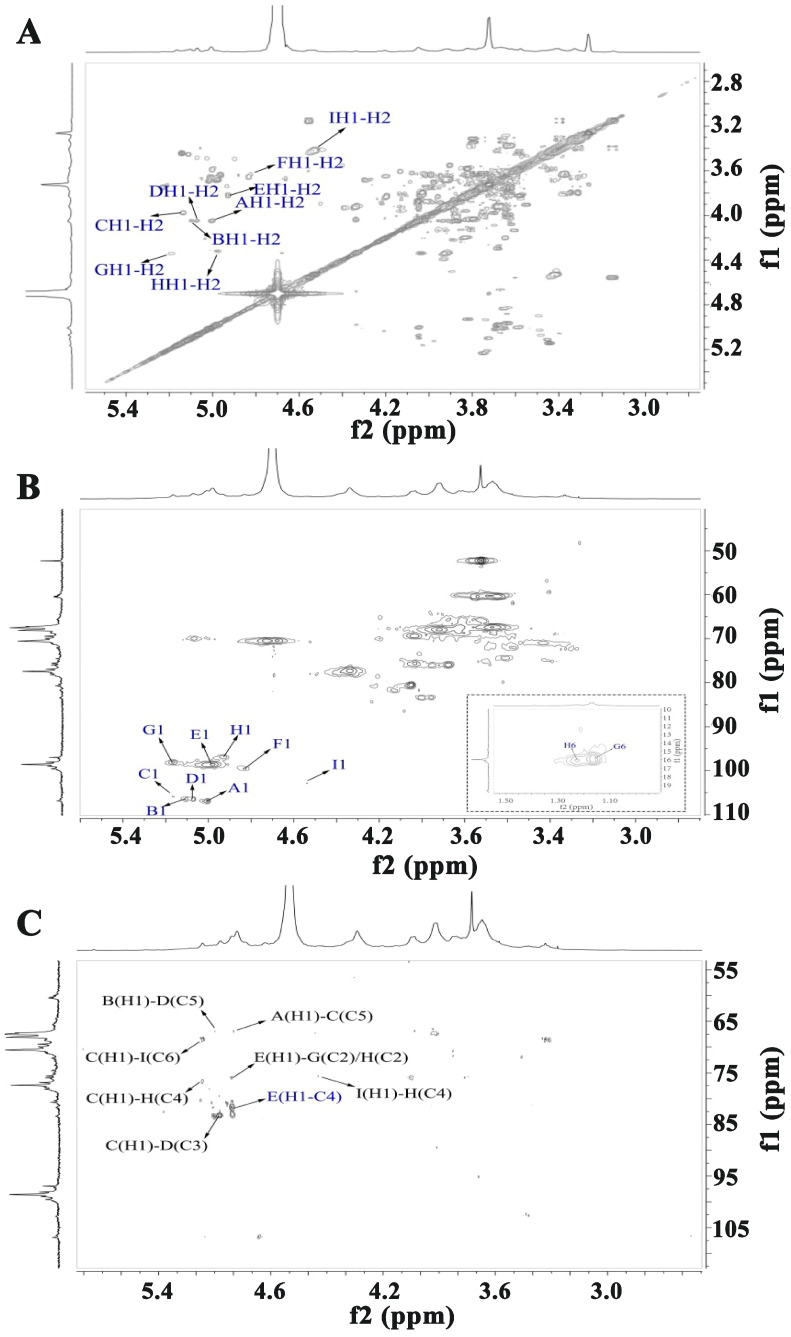
Structural elucidation of WATP-A3b by 2D NMR: (**A**) COSY spectrum, (**B**) HSQC spectrum, and (**C**) HMBC spectrum (residue designations A–I correspond to chemical shift data in Table 3).

Figure 5 and Figure 6 present the NMR spectral analysis of WATP-A3b. The ^1^H NMR spectrum (Figure 5A) exhibits anomeric proton resonances from 4.56 to 5.16 ppm, with corresponding anomeric carbon signals in the ^13^C NMR spectrum (Figure 5B) appearing between 96.9 and 106.92 ppm, demonstrating the coexistence of *α*- and *β*-configured sugar residues. GalA acetylation is confirmed by signals at 3.73 ppm (^1^H NMR) and 19.45 ppm (^13^C NMR), while the strong ^13^C NMR signal at 174.45 ppm reveals predominantly demethylated GalA residues. Similar to WATP-A2b, nine major signals were also observed in the ^1^H-^13^C HSQC spectrum of WATP-A3b, specifically at 5.00/106.92 (A), 5.11/106.34 (B), 5.16/105.85 (C), 5.07/106.49 (D), 4.98/98.60 (E), 4.83/99.53 (F), 5.16/98.11 (G), 4.93/96.90 (H), and 4.56/103.23 ppm (I). These findings suggest that WATP-A3b primarily contains nine different sugar residues. Through comparative analysis of monosaccharide composition and reference chemical shift data, complete ^1^H and ^13^C NMR assignments for WATP-A3b residues were established. As detailed in Table 3, residues A-I were identified as *α*-Ara*f-*(1→^AtI^, *α*-Ara*f-*(1→^AtII^, (1→5)-*α*-Ara*f*, (1→3,5)-*α*-Ara*f*, (1→4)-*α*-GlaA*p*, (1→4)-*α*-GlaA*p*6Me, (1→2)-*α*-Rha*p*, (1→2,4)-*α*-Rha*p* and (1→3,6)-*β*-Gal*p*.

HMBC spectroscopy was applied to investigate the inter-residue connectivity of structural units A-I; signals at 5.01/81.69 ppm and 5.01/75.91 ppm were observed, representing the H-C long-range coupling peaks of residues EH1-EC4 and EH1-GC2/HC2, respectively. These results indicate that WATP-A3b contains [→4)-GalA*p*-(1→4)-GalA*p*(1→], [→4)-*α*-GalA*p*-(1→2)-*α*-Rhap-(1→], and [→4)-*α*-GalA*p*-(1→2,4)-*α*-Rha*p*-(1→] structural units, suggesting the presence of HG and RG-I domains. The signals at 5.16/76.54 ppm (CH1-HC4) and 4.56/76.54 ppm (IH1-HC4) in the HMBC spectrum indicate that the WATP-A3b side chain connects to C4 of *α*-Rha*p* in the RG-I backbone through (1→3,6)-*β*-Gal*p* and (1→5)-*α*-Ara*f* residues. Additionally, signals at 5.00/67.06 ppm (AHI-CC5), 5.16/68.32 ppm (CH1-IC6), 5.00/67.06 ppm (BH1-DC5), and 3.16/68.32 ppm (CH1-DC3) were detected in the HMBC spectrum. These results suggest that WATP-A3b contains a short chain comprising [t-*α*-Ara*f*-(1→5)-*α*-Ara*f*-(1→] structural units and an AG-II domain comprising (1→5)-*α*-Ara*f*, (1→3,5)-*α*-Ara*f*, (1→3,6)-*β*-Gal*p,* and t-*α*-Ara*f* residues. Therefore, similar to WATP-A2b, the backbone of WATP-A3b consists of HG and RG-I domains. However, its side-chain composition and the connection mode between the side chain and the backbone differ from WATP-A2b. Specifically, WATP-A3b contains a short chain and an AG-II domain composed of t-*α*-Ara*f* and (1→5)-*α*-Ara*f*, which are anchored to the RG-I backbone via (1→5)-*α*-Ara*f* and (1→3,6)-*β*-Gal*p*, respectively.

### 2.5. Advanced Conformational Analysis of WATP-A2b and WATP-A3b

#### 2.5.1. Congo Red Assay of WATP-A2b and WATP-A3b

**Figure 7:**
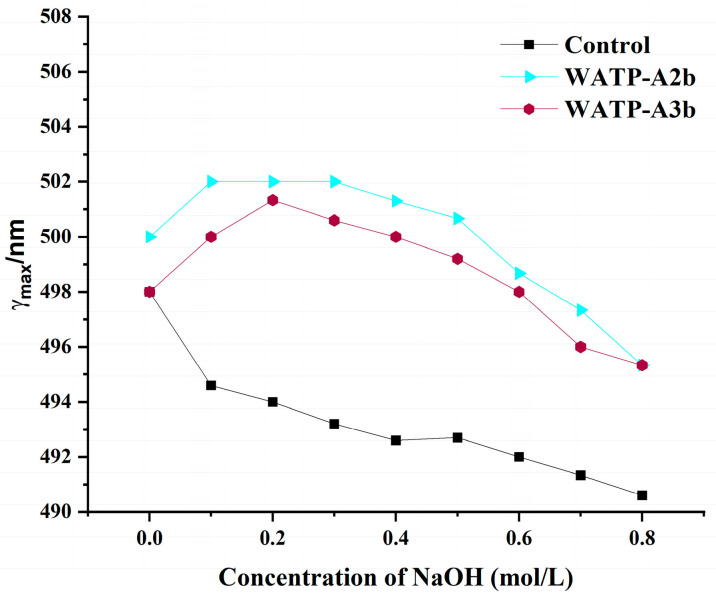
Variation in absorption wavelength maxima of pectin–Congo red complexes with increasing sodium hydroxide concentration.

The formation of Congo red–polysaccharide complexes provides a method for detecting helical structures. These complexes demonstrate increased absorption wavelengths in sodium hydroxide (NaOH) solutions compared to free Congo red. When NaOH concentration reaches 0.3 M, the disruption of hydrogen bonds destabilizes the helical conformation, resulting in a marked reduction in the absorption maximum. This characteristic spectral behavior allows for the identification of triple-helical polysaccharide conformations through visible light spectroscopy [27]. Figure 7 illustrates the absorption wavelength trends of WATP-A2b and WATP-A3b in NaOH solutions (0–0.8 M). The Congo red control shows a consistent reduction in absorption maximum within the 0.05–0.8 M range. Both pectin fractions form stable Congo red complexes, with their absorption maxima displaying an initial rise followed by a decline. The absorbance of both WATP-A2b and WATP-A3b peaks when the NaOH solution concentration is 0.2 M. With further increases in the solution, the maximum wavelength gradually decreases. In conclusion, both WATP-A2b and WATP-A3b contain a triple-helix structure.

#### 2.5.2. Circular Dichroism (CD) Analysis of WATP-A2b and WATP-A3b

**Figure 8:**
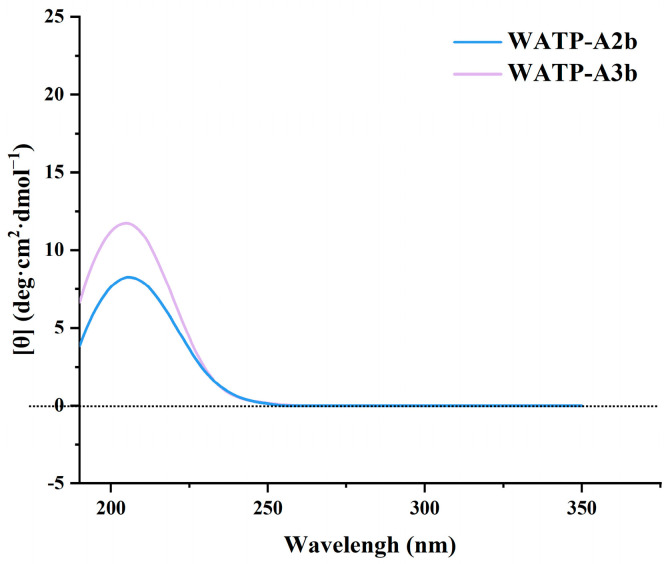
CD spectra of WATP-A2b and WATP-A3b.

CD spectroscopy serves as a valuable tool for quickly assessing secondary structure and local tertiary conformations in biomacromolecules. The presence of uronic acid, acetate, and other luminescent groups in pectin has enabled the exploration of pectin conformations and their conformational transitions through CD [28]. In this study, CD technology was used to compare and analyze the conformations of WATP-A2b and WATP-A3b, with molar ellipticity [θ] as the ordinate and wavelength as the abscissa. The results are presented in Figure 8. At 205 nm, both WATP-A2b and WATP-A3b exhibited obvious positive phase peaks. A prominent positive Cotton effect, observed around 210 nm, arises from n-π* transitions in GalA carboxyl groups within the pectin structure [28]. The findings of this study align with those of Qiu et al. and Nitta et al. [29,30]. Qiu et al. [29] observed a similar CD spectrum for citrus pectin, showing a strong positive single peak at 202 nm. Nitta et al. [30] found that spiral pectin also displayed a strong positive single peak at 202 nm. The experimental results indicated that both WATP-A2b and WATP-A3b possessed helical conformation regions, which is consistent with the experimental results obtained using Congo red.

Table 4 shows that the [θ] values of WATP-A2b and WATP-A3b are 8.26 deg·cm^2^·dmol^−1^ and 11.74 deg·cm^2^·dmol^−1^, respectively. Variations in the 202 nm ellipticity peak reflect changes in hydrogen bonding interactions. Reduced intramolecular hydrogen bonding leads to diminished ellipticity at this characteristic wavelength [29]. The significant difference in CD peak height between WATP-A2b and WATP-A3b reflects that these two pectins have distinct spatial conformations, implying that WATP-A2b and WATP-A3b may exhibit a more flexible chain conformation. The asymmetric structure of polysaccharides and the degree of structural asymmetry can influence the peak position and intensity of the Cotton effect in CD [31]. The [θ] values of WATP-A2b and WATP-A3b were positively correlated with their GalA content.

#### 2.5.3. Scanning Electron Microscopy (SEM) Analysis of WATP-A2b and WATP-A3b

**Figure 9:**
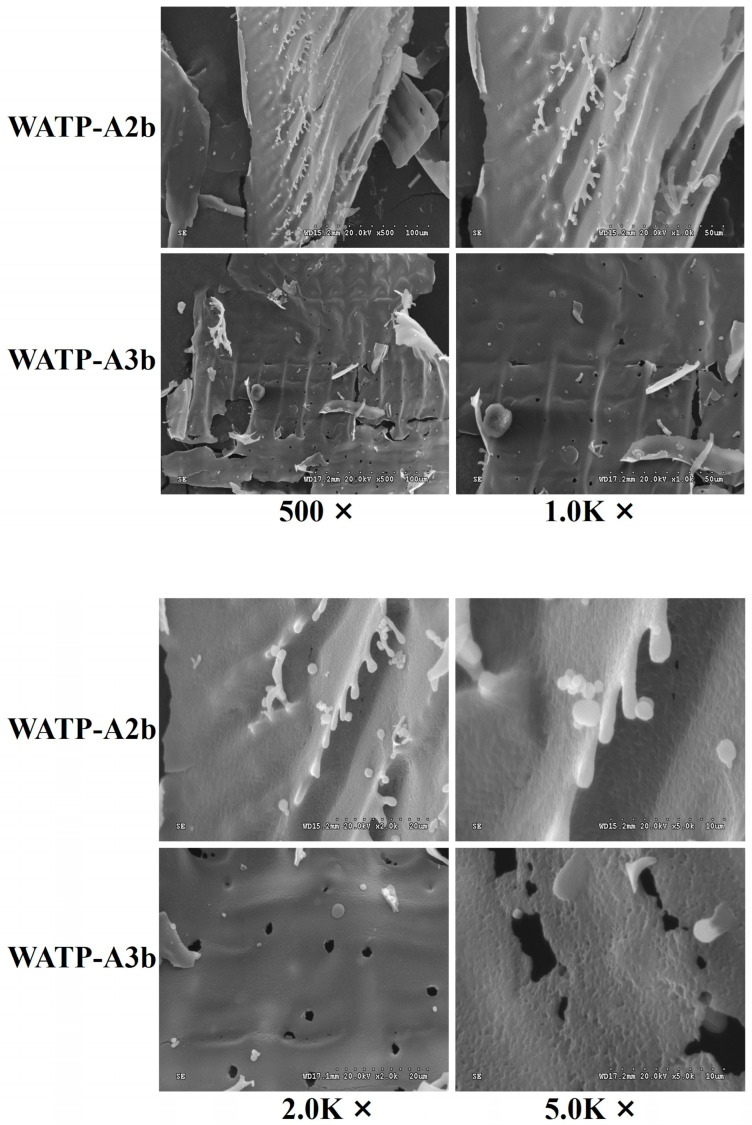
Scanning electron micrographs of WATP-A2b and WATP-A3b.

As a versatile imaging tool, SEM facilitates detailed analysis of polysaccharide surface morphology and structural organization, delivering direct evidence of microstructural features. This includes insights into the shape, porosity, and molecular size of polysaccharides and other macromolecules [32]. In this study, we analyzed the morphology of WATP-A2b and WATP-A3b using SEM. The SEM results at magnifications of 500, 1000, 2000, and 5000 are depicted in Figure 9. During preliminary microscopic examination, WATP-A2b exhibits a folded lamellar structure characterized by smooth lamellae with irregularly distributed short filamentous extensions (approximately 10 μm in diameter) at the edges, accompanied by microspores of similar diameter. In contrast, WATP-A3b displays a more uniform and orderly lamellar structure during preliminary microscopic examination, which reveals an uneven surface with irregularly scattered pores at 5000× magnification, devoid of edge filamentous extensions. These structural distinctions likely originate from variations in molecular size, carbohydrate composition, and the distribution of HG, RG, and AG structural domains. The observed porosity in WATP-A3b can be attributed to multiple factors: (1) the inherent molecular arrangement during polysaccharide self-assembly creating natural void spaces, (2) the spatial distribution of structural domains with varying hydrophilicity contributing to micro-pore formation, and (3) potential artifacts from sample preparation, particularly water evaporation during vacuum drying. The irregular pore distribution at high magnification reflects WATP-A3b’s heterogeneous composition, including molecular weight distribution and branching pattern variations, suggesting fundamental differences in molecular organization and physicochemical properties compared to WATP-A2b.

### 2.6. Enzymatic Analysis of WATP-A2b and WATP-A3b

Compositional and NMR studies demonstrate that WATP-A2b and WATP-A3b contain HG, RG-I, and AG-II domains, along with arabinan side chains. To better understand their structural and functional properties, endo-polygalacturonase (endo-PG) (EC 3.2.1.15) was utilized for enzymatic hydrolysis, followed by domain separation via HPGPC.

#### 2.6.1. Preparation of De-Esterified Pectin

**Figure 10:**
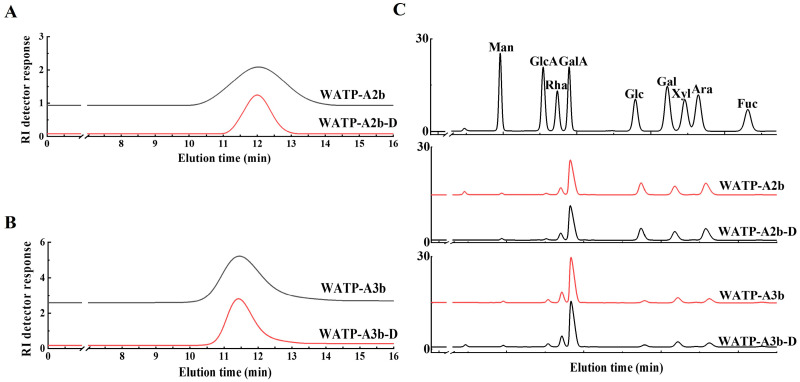
Analytical characterization of de-esterified products WATP-A2b-D and WATP-A3b-D. (**A**,**B**) HPGPC elution profiles; (**C**) monosaccharide distribution.

The endo-PG enzyme exhibits specificity for non-esterified GalA residues within HG domains, though methyl and acetyl group presence can inhibit enzymatic activity, potentially affecting HG domain purity [33]. This study implemented mild alkaline treatment at lower temperatures to optimize pectin de-esterification. Structural preservation and de-esterification efficiency were evaluated through FT-IR, HPGPC, and monosaccharide composition analysis. HPGPC elution patterns (Figure 10A,B) revealed unchanged molecular weight profiles after saponification, indicating intact pectin chains. Monosaccharide composition analysis (Figure 10C) showed minimal differences between native and de-esterified fractions. FT-IR spectra (Figure 1E,F) demonstrated elimination of the 1740 cm^−1^ methyl ester peak and enhanced intensity at 1610 cm^−1^, verifying effective methyl group removal.

#### 2.6.2. Analysis of Enzymatic Hydrolysates

**Figure 11:**
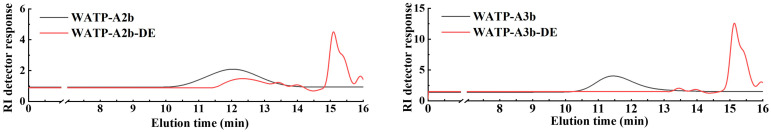
The HPGPC elution patterns of both pectin samples and their enzymatically de-esterified hydrolysates.

Endo-PG specifically hydrolyzes non-esterified GalA bonds, breaking down HG domains into oligogalacturonides and releasing RG-I and RG-II domains. This research employed endo-PG to digest both pectin samples, yielding WATP-A2b-DE and WATP-A3b-DE hydrolysates. HPGPC analysis indicated substantial molecular weight modifications, as shown by multiple chromatographic peaks (Figure 11). Additional purification via Sephadex G-75 chromatography produced three distinct hydrolysate fractions (E1–E3) from each pectin type.

The molecular weights of WATP-A2b-DE1 and WATP-A3b-DE1, determined as 31.1 kDa and 45.3 kDa, respectively (Table 5), revealed distinct structural characteristics. Both de-esterified hydrolysates were primarily composed of GalA, Rha, Gal, and Ara, with a Rha/GalA molar ratio close to 1.0, confirming their classification as RG-I-type pectins. However, WATP-A2b-D-E1 exhibited significantly higher proportions of Gal (22.6%) and Ara (37.3%) compared to WATP-A3b-D-E1. The (Gal + Ara)/Rha ratio, a key indicator of neutral side-chain length and composition within the RG-I domain [33], was 3.6 for WATP-A2b-D-E1, approximately 3.6-fold greater than that of WATP-A3b-D-E1. This suggests that WATP-A2b-DE1 possesses either longer or more highly branched neutral sugar side chains. Both WATP-A2b-D-E2 and WATP-A3b-D-E2 tested positive in TBA tests, confirming their classification as RG-II pectins. Additionally, the molecular weight distribution of WATP-A2b-D-E2 and WATP-A3b-D-E2 was heterogeneous, with both showing double peaks on HPGPC. Additionally, WATP-A2b-D-E3 and WATP-A3b-D-E3 exhibited molecular weights under 2.0 kDa, with their hydrolysates predominantly consisting of GalA (95.3–96.6%). This composition indicates that these compounds are oligogalacturonides, likely produced through the endo-PG-mediated breakdown of HG-type pectin regions.

### 2.7. Assessment of Antioxidant Capacity

**Figure 12:**
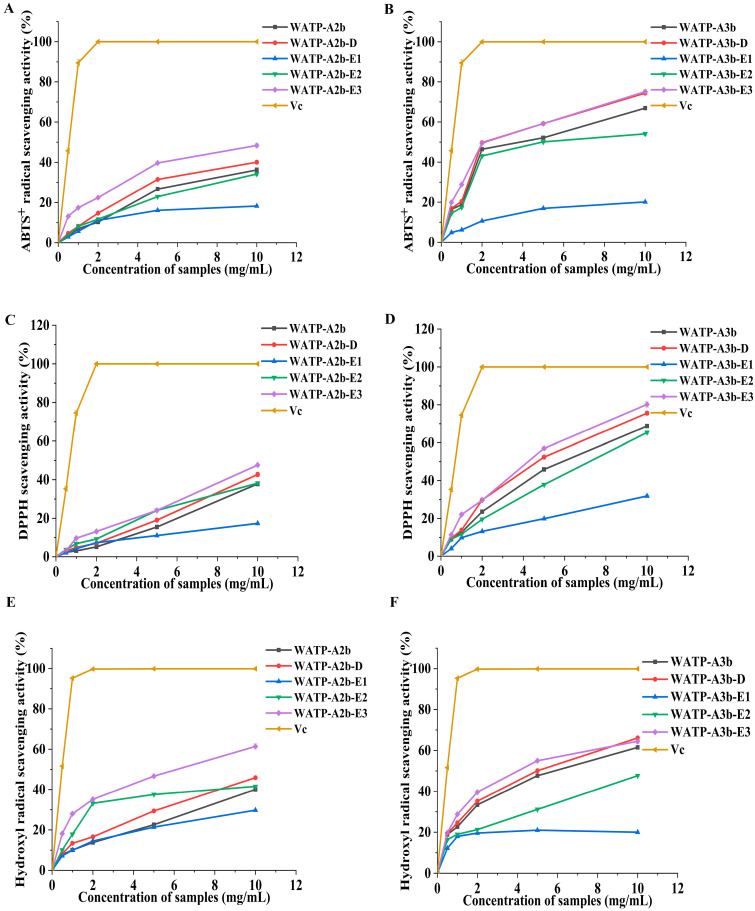
Scavenging effects of *Adenophora tetraphylla* pectin fractions on (**A**,**B**) ABTS, (**C**,**D**) DPPH, and (**E**,**F**) hydroxyl radicals. *L*-Ascorbic acid was used as the reference. Values are expressed as mean ± standard deviation (*n* = 3).

The in vitro antioxidant properties of WATP-A2b, WATP-A3b, their de-esterified derivatives, and enzymatic hydrolysates were investigated by measuring their ABTS, DPPH, and hydroxyl radical scavenging activities. At concentrations ranging from 0.5 to 10 mg/mL, all samples displayed a notable dose-dependent inhibition of these radicals, as depicted in Figure 12. Notably, WATP-A2b exhibited IC_50_ values of 17.58, 13.93, and 18.63 mg/mL against the three radicals, respectively, while WATP-A3b showed significantly lower IC_50_ values of 3.89, 5.40, and 5.40 mg/mL. These results indicate that WATP-A3b possessed a higher radical scavenging ability compared to WATP-A2b, although its activity remained inferior to that of *L*-ascorbic acid.

To further explore the relationship between antioxidant activity and structural characteristics of WATP-A2b and WATP-A3b, the radical scavenging capacities of their de-esterified forms (WATP-A2b-D and WATP-A3b-D) and enzymatic hydrolysates were assessed. The degree of methyl-esterification plays a significant role in modulating the antioxidant properties of pectins, with methylation levels being a critical factor influencing their activity. Previous research has demonstrated an inverse correlation between the antioxidant efficacy of apple pectins and their methylation degree [34].

In this investigation, WATP-A3b exhibited superior ABTS, DPPH, and hydroxyl radical scavenging capabilities compared to WATP-A2b, even though the latter had a higher methyl-esterification degree. Following de-esterification, both WATP-A2b-D and WATP-A3b-D displayed markedly enhanced antioxidant performance relative to their native forms. These findings highlight the intricate interplay between pectin structure and antioxidant functionality, which is likely governed by multiple contributing factors. Moreover, molecular weight is a key determinant of polysaccharide antioxidant activity. High-molecular-weight pectins tend to form extensive hydrogen bonds, both intermolecularly and intramolecularly, which can limit the availability of hydroxyl groups. In contrast, lower-molecular-weight pectins often adopt a more open conformation, facilitating the exposure of free hydroxyl groups and thereby improving radical scavenging efficiency [35]. Additionally, the antioxidant activity of polysaccharides is significantly influenced by their monosaccharide composition. Research has shown that GlcA and GalA play a crucial role in enhancing the radical scavenging capacity of *Cissus pteroclada*, particularly against DPPH, ABTS, hydroxyl, and superoxide radicals [36].

Pectins with a significant proportion of GalA are well known for their potent antioxidant properties, which are closely associated with their uronic acid content and degree of polymerization [37]. Among the tested fractions, oligogalacturonides (E3), characterized by the highest GalA levels and the lowest molecular weight, exhibited the strongest radical scavenging activity, outperforming the RG-II domain (E2). In contrast, the RG-I domain (E1), with its reduced GalA content and higher branching, displayed the lowest scavenging capacity. Importantly, the radical scavenging efficacy of oligogalacturonides increased in a concentration-dependent manner and exceeded that of the native pectins (WATP-A2b and WATP-A3b) at equivalent concentrations. These findings align with prior studies, further supporting the notion that pectins rich in GalA and containing HG-type domains possess superior radical scavenging potential [38].

The findings suggest that WATP-A3b exhibits greater antioxidant activity than WATP-A2b, likely attributed to its elevated GalA content. However, its higher molecular weight and methyl-esterification levels appear to hinder its free radical scavenging efficiency. The in vitro antioxidant behavior of both WATP-A2b and WATP-A3b arises from the synergistic contributions of different pectin domains, with the HG domain playing the most prominent role, followed by the RG-II domain. On the other hand, the RG-I domain, which features increased branching and a larger molecular size, contributed the least to the overall antioxidant performance of the pectins.

### 2.8. Discussion

The comprehensive structural characterization and antioxidant evaluation of WATP-A2b and WATP-A3b from *Adenophora tetraphylla* provide significant insights into the structure–activity relationships of pectic polysaccharides. This study represents a notable advancement in the field for several reasons. Firstly, while the pectin content of *Adenophora tetraphylla* has been previously measured [17], this work constitutes the first systematic investigation of the structural characteristics and antioxidant capacity of pectins from this medicinal plant, which has a long history of use in traditional Chinese medicine but has remained largely unexplored in terms of its pectin composition and bioactivity. The identification and characterization of two distinct pectin fractions with varying structural domains and bioactivities contribute novel data to the growing body of knowledge on plant-derived polysaccharides.

The observed differences in antioxidant activity between WATP-A2b and WATP-A3b can be attributed to several structural factors. The higher antioxidant capacity of WATP-A3b is likely due to its elevated GalA content compared to WATP-A2b, which provides more carboxyl groups for radical scavenging [39]. Additionally, the lower degree of methylation in WATP-A3b enhances its solubility and accessibility to free radicals, further contributing to its superior performance [34]. In contrast, the presence of a more extensive RG-I domain in WATP-A2b may hinder its antioxidant activity, as the branched structure could limit the exposure of active groups [40]. These findings align with previous studies on pectin structure–activity relationships [39,40,41] while providing unique insights specific to *Adenophora tetraphylla* pectins.

The potential medical applications of these pectins are supported by their demonstrated bioactivities and favorable safety profile. The strong antioxidant properties, particularly of WATP-A3b, suggest potential use in the following areas: (1) Antioxidant therapy: WATP-A2b and WATP-A3b could be utilized as natural antioxidant supplements to mitigate oxidative stress-related conditions, such as chronic inflammation, cardiovascular diseases, and neurodegenerative disorders, as supported by numerous studies on pectin [7]. (2) Gastrointestinal protection: Pectin has been shown to prevent and treat gastric ulcers and inflammatory bowel disease through its antioxidant and anti-inflammatory effects, indicating that WATP-A2b and WATP-A3b may have therapeutic potential for these conditions [42,43]. (3) Wound healing: The ability of pectin to scavenge free radicals and regulate oxidative stress positions it as a promising candidate for wound healing applications, suggesting that WATP-A2b and WATP-A3b could also be explored for such uses.

While this study provides valuable insights into the structural characteristics and antioxidant capacity of pectins from *Adenophora tetraphylla*, some limitations should be noted. The extraction and purification processes may not fully capture the structural diversity of pectins, potentially overlooking minor but biologically significant fractions. Additionally, the in vitro antioxidant assays may not fully replicate the complex biological environments in vivo, limiting the extrapolation of results. Future research should focus on in vivo studies, alternative extraction methods, and a deeper exploration of structure–activity relationships to better understand and optimize the therapeutic potential of these pectins.

## 3. Materials and Methods

### 3.1. Materials

In August 2023, the roots of *Adenophora tetraphylla* were collected (voucher specimen number: 2023-08-20-001) from Tonghua, Jilin Province, China, ensuring uniformity by sourcing from plants in the same region. The authenticity of the specimens was confirmed by Professor Junlin Yu, and a voucher specimen has been preserved in the Herbarium of Engineering Research Center of Glycoconjugates, Ministry of Education. Prior to processing, the root material was minimally prepared through cleaning and washing, followed by slicing and air-drying in a cool, ventilated environment for subsequent use. DEAE cellulose and Sepharose CL-6B were procured from GE Healthcare (Chicago, IL, USA), and monosaccharide standards were sourced from Sigma-Aldrich (St. Louis, CA, USA). All other reagents used in the study were of analytical grade.

### 3.2. Methods

The total carbohydrate content was quantified using the phenol–sulfuric acid method, with primary monosaccharides serving as the standard, as detailed in [44]. Additionally, the uronic acid content was analyzed via the *m*-hydroxydiphenyl method, employing GalA as the reference, following the procedure described in [44]. Absorbance measurements were conducted using a UV-2700 UV scanner (Shimadzu, Kyoto, Japan), covering a spectrum of 200 to 800 nm. Homogeneity and molecular weight were evaluated using a HPLC system (Shimadzu, Kyoto, Japan), equipped with an RID-20A UV detector (Shimadzu, Kyoto, Japan) and a TSKgel G3000PWXL column (Tosoh, Tokyo, Japan). The TBA method, as outlined in [44], was utilized for the detection of 3-deoxy-*D*-manno-2-octulosonic acid.

### 3.3. Pectin Preparation from Adenophora tetraphylla

#### 3.3.1. Pectin Extraction Process

The *Adenophora tetraphylla* material was extracted using a hot-water method, following the procedure detailed in reference [44]. Briefly, 1 kg of dried material was ground and immersed in 16 L of deionized water. Extraction was performed at 100 °C for 3 h, with the process repeated three times under identical conditions. The supernatant was then concentrated to 2 L at 80 °C and mixed with 8 L of 95% ethanol to induce precipitation. The mixture was left to stand overnight at 4 °C. The precipitates were washed successively with 95% ethanol and anhydrous ethanol, followed by vacuum drying at 60 °C overnight. The resulting crude polysaccharide, designated as WATP, was obtained from the *Adenophora tetraphylla* material.

#### 3.3.2. Pectin Fractionation

A total of 50 g of WATP was completely dissolved in 1 L of deionized water. The solution was centrifuged and applied to a DEAE–cellulose preparative column (Cl-type). After a 30 min equilibration period, the crude polysaccharide was first eluted with 4.5 L of deionized water. The eluate was concentrated and freeze-dried to produce the neutral fraction, designated as WATP-N. The column was then eluted with 3 L of 0.5 M NaCl solution to isolate the crude pectin fraction, referred to as WATP-A. The yields of both fractions were measured and calculated.

**Figure 13:**
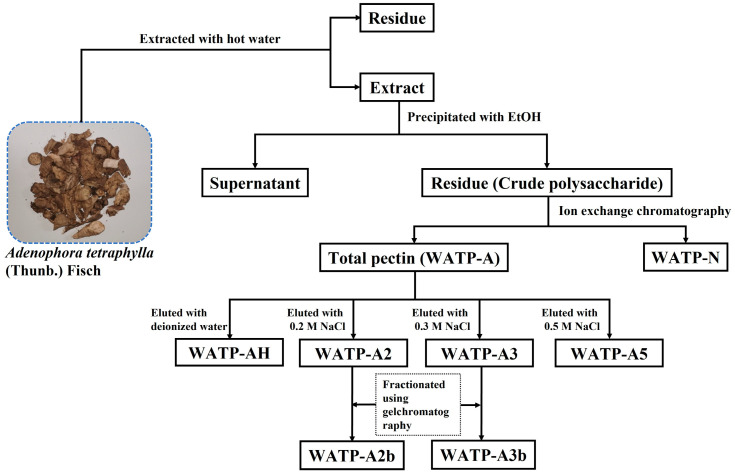
The process of isolating and purifying the pectins WATP-A2b and WATP-A3b from *Adenophora tetraphylla*.

A total of 1 g of WATP-A was completely dissolved in distilled water, centrifuged, and applied to a DEAE–cellulose column. The raw pectin was sequentially eluted using deionized water and 0.2 M, 0.3 M, and 0.5 M NaCl solutions at a flow rate of 25 mL/min. Based on the total carbohydrate and uronic acid content analysis of the eluted fractions, they were identified and labeled as WATP-AH, WATP-A2, WATP-A3, and WATP-A5. To achieve further purification, WATP-A2 and WATP-A3 were passed through a Sepharose CL-6B column and eluted with a 0.15 M NaCl solution at 0.5 mL/min. This yielded two purified pectic polysaccharides, designated as WATP-A2b and WATP-A3b. Figure 13 provides a summary of the extraction and fractionation method used to obtain pectins from *Adenophora tetraphylla*.

### 3.4. Chemical Characterization Analysis

A polysaccharide sample (2–4 mg) was hydrolyzed using a 2 M anhydrous HCl–methanol solution and trifluoroacetic acid, following established methods [44]. The hydrolyzed product was then derivatized with PMP at 70 °C for 30 min. The PMP derivatives were purified via chloroform extraction and analyzed using an HPLC system equipped with an SPD-20A UV-Vis diode-array detector (Shimadzu, Kyoto, Japan) and a COSMOSIL 5C18-PAQ column (Nacalai Tesque, Kyoto, Japan). The mobile phase consisted of 0.1 M PBS (pH 6.9) with 17% acetonitrile (*v*/*v*). The analysis conditions included a column temperature of 35 °C, a detection wavelength of 245 nm, a flow rate of 1 mL/min, and an injection volume of 10 μL.

### 3.5. FT-IR Analysis

Completely dried samples (2 mg each) were blended with KBr at a 1:100 weight ratio and examined using a Spectrum Two FT-IR spectrometer (PerkinElmer, Waltham, MA, USA). The FT-IR spectra were recorded over a range of 4000 to 400 cm^−1^. Spectra acquisition and pre-processing were performed using Spectrum 10™ software (version 10.5.3, PerkinElmer, Waltham, MA, USA).

### 3.6. NMR Analysis

For NMR characterization, 20 mg of each sample was dissolved in 0.5 mL of 99.9% D_2_O. The spectra, including ^1^H NMR, ^13^C NMR, ^1^H-^1^H COSY, ^1^H-^13^C HSQC, and HMBC, were acquired at 25 °C using a AV600 MHz NMR spectrometer (Bruker, Billerica, MA, USA).

### 3.7. Congo Red Analysis

Prepare a 2.5 mg/mL sample solution in distilled water. Combine 2 mL of this solution with an equal volume of 80 μM Congo red solution. Add 1 mL of NaOH solution at varying concentrations (0–0.8 M, in 0.1 M increments) to achieve the target NaOH levels in the final mixture. After incubating at room temperature for 30 min, measure the absorbance across the 400–700 nm range [45].

### 3.8. CD Analysis

We dissolved 2 mg of the sample in deionized water to create a 2 mg/mL solution. The sample’s conformation was then analyzed using a CD Spectroscope (Applied Photophysics, Leatherhead, UK). The detection parameters included a wavelength range of 190–350 nm, a scanning bandwidth of 2.5 nm, a time constant of 2.0 s, and a scanning rate of 20 nm/min. Each measurement was performed in triplicate, and the average value was calculated to ensure accuracy [46]. 

### 3.9. SEM Analysis

Sample preparation and SEM analysis were conducted following established protocols [30]. Briefly, 5–10 mg of sample was vacuum-dried at 45 °C overnight. The dried sample was then mounted on the sample stage using conductive adhesive and subjected to conductivity treatment using an SB-2 ion-sputtering gold coater (Beijing Zhongke, Beijing, China). Gold coating was performed under low-vacuum mode for 4–5 min at an operating current of 5–6 mA. The coated samples were subsequently analyzed using a S-3000N SEM (Hitachi, Tokyo, Japan) operated in high-vacuum mode at 20 kV accelerating voltage.

The image acquisition parameters were carefully optimized to ensure accurate morphological characterization. Secondary electron images were acquired at 5.0 nm resolution under the following conditions: 20 μs/pixel dwell time, 8-frame accumulation, 10 frames/s scan speed, and 120 s total acquisition time per image. These settings provided an optimal balance between image quality and sample preservation, maintaining sufficient signal-to-noise ratio while minimizing potential beam damage. All SEM images were processed and analyzed using PC-SEM 2.0 software.

### 3.10. De-Esterification and Enzymatic Degradation

To prepare de-esterified pectins, 500 mg of WATP-A2b and WATP-A3b were separately dissolved in 15 mL of distilled water. The solutions were mixed thoroughly and pre-cooled at 4 °C for 6 h. Subsequently, 15 mL of pre-cooled 0.2 M NaOH solution was slowly added to each sample, and the mixtures were stirred gently at 4 °C for 4 h [44]. The reaction solutions were neutralized to pH 7.0 using 10% acetic acid, desalted via a Sephadex G-10 column (Cytiva, Marlborough, MA, USA), and freeze-dried to obtain the de-esterified pectins, labeled as WATP-A2b-D and WATP-A3b-D.

For enzymatic hydrolysis, WATP-A2b-D and WATP-A3b-D were dissolved in a 50 mM acetic acid–ammonium acetate buffer (pH 5.0) at a concentration of 1 mg/mL. Then, 50 μL of endo-PG was added, and the mixture was incubated at 40 °C for 24 h. The reaction was terminated by heating in a boiling water bath for 15 min. The hydrolysates were fractionated using a Sephadex G-75 column (Cytiva, Marlborough, MA, USA) eluted with 0.15 M NaCl at 0.4 mL/min. The eluted fractions were collected, desalted through a Sephadex G-10 column, and freeze-dried. Three subfractions were obtained from each de-esterified pectin, designated as WATP-A2b-D-E1 to E3 and WATP-A3b-D-E1 to E3, respectively.

### 3.11. Evaluation of Antioxidant Activity

#### 3.11.1. ABTS Radical Scavenging Assay

The ABTS radical scavenging activity of the pectin fractions was evaluated following a previously described method [44]. A fresh ABTS working solution was prepared daily. For the assay, 400 μL of each sample solution (0.5–10 mg/mL) was mixed with 400 μL of the ABTS working solution in a reaction tube. The mixture was incubated in the dark at 25 °C for 30 min, after which the absorbance was measured at 732 nm. Ultrapure water was used as the control in place of the sample solutions. The scavenging activity was calculated using the specified formula.

$$\%scavenging=\left(1-\frac{A_{sample}-A_{control}}{A_{blank}}\right)\times 100\%$$

A_sample_: This is the absorbance value measured for the sample solution.
A_control_: This is the absorbance value measured for the background solution.
A_blank_: This is the absorbance value measured for the blank control.

#### 3.11.2. DPPH Radical Scavenging Assay

The DPPH radical scavenging capacity of the pectin fractions was assessed according to established protocols [44]. Briefly, 500 μL of each pectin fraction (0.5–10 mg/mL) was mixed with 2 mL of 0.5 mM DPPH solution. The mixture was kept in the dark for 30 min, and the absorbance was measured at 517 nm. *L*-ascorbic acid served as the positive control, while ultrapure water and anhydrous methanol were used as blank controls. The scavenging activity was calculated using the same formula applied in the ABTS assay.

#### 3.11.3. Hydroxyl Radical Scavenging Assay

The hydroxyl radical scavenging activity of the pectin fractions was measured following a previously described method [44]. In this assay, 100 μL of pectin solution (0.5–10 mg/mL) was mixed with 100 μL of 9.0 mM FeSO_4_ solution and 100 μL of 9.0 mM salicylic acid solution in absolute ethanol. The mixture was then combined with 100 μL of 8.8 mM H_2_O_2_ solution and incubated at 25 °C for 30 min. Absorbance was recorded at 532 nm. Ultrapure water replaced the pectin solution as the control, while *L*-ascorbic acid was used as the positive control. A secondary control was prepared by substituting the H_2_O_2_ solution with ultrapure water. The scavenging activity was calculated using the same formula as in the ABTS assay.

### 3.12. Statistical Analysis

The antioxidant activity results are expressed as mean ± standard deviation. Statistical differences between groups were determined using one-way ANOVA, followed by LSD post hoc tests. All experiments were performed in triplicate to ensure reproducibility. Data analysis was conducted using SPSS software (version 23.0, IBM, Armonk, NY, USA).

## 4. Conclusions

This study aimed to comprehensively investigate the structural properties and antioxidant activities of two pectins, WATP-A2b and WATP-A3b, extracted from *Adenophora tetraphylla*. These pectins, purified through a multi-step process, have molecular weights of 22.5 kDa and 49.8 kDa, respectively. Their monosaccharide composition is dominated by GalA, Rha, Gal, Ara, and Glc, which collectively constitute over 93% of their total content. Structural analysis identified three distinct domains—HG, RG-I, and RG-II—with varying mass ratios. Both pectins are primarily composed of RG-I domains, with WATP-A2b containing 61.6% and WATP-A3b containing 53.4%. FT-IR spectroscopy was employed to examine their primary structure and methylation degree, while 1D and 2D NMR techniques elucidated the intricate linkage patterns of their sugar residues. These findings were corroborated by Congo red, CD, and SEM analyses. Further examination of the de-esterified derivatives and enzymatic hydrolysates of WATP-A2b and WATP-A3b provided deeper insights into their structural features. Notably, the two pectins exhibited significant differences in methyl esterification levels, which influenced their radical scavenging capacities. WATP-A3b, with its higher GalA content, demonstrated superior antioxidant activity against DPPH, ABTS, and hydroxyl radicals, positioning it as a promising natural antioxidant with potential pharmaceutical applications.

## Figures and Tables

**Table 1:** Extraction yield, molecular characteristics, and carbohydrate profile of pectins isolated from *Adenophora tetraphylla*.

	WATP	WATP-N	WATP-A	WATP-A2b	WATP-A3b
Yield (*w*%)	5.4 ^a^	43.1 ^b^	23.5 ^b^	16.1 ^c^	23.1 ^c^
Molecular weight (kDa)		ND	ND	22.5	49.8
**Monosaccharide composition**					
GalA	25.4	2.5	45.0	34.4	54.0
Rha	6.5	1.1	8.1	12.4	15.8
Gal	16.2	7.3	14.7	12.9	10.1
Ara	23.2	19.0	18.8	23.9	11.7
Glc	19.3	66.9	7.8	13.4	2.0
GlcA	3.6	0.8	2.1	1.2	2.7
Xyl	1.2	0.6	1.7	NONE	0.8
Man	3.6	1.6	1.8	1.3	2.2

^a^ Yield based on dry weight of the plant material. ^b^ Yield based on WATP. ^c^ Yield based on WATP-A. ND: Not detected. WATP-N: Water-soluble *Adenophora tetraphylla* polysaccharide—neutral. WATP-A: Water-soluble *Adenophora tetraphylla* polysaccharide—acidic.

**Table 2:** ^1^H and ^13^C NMR spectral data assignments of WATP-A2b structural units.

Residues	Glycosidic Linkage		1	2	3	4	5	6
A	*α*-Ara*f-*(1→^tI^	H	5.02	4.01	3.88	4.06	3.65	
		C	106.86	83.34	77.33	80.49	60.43	
B	*α*-Ara*f-*(1→^tII^	H	5.11	4.01	3.88	4.06	3.75	
		C	106.47	83.34	77.328	80.49	60.38	
C	→5)-*α*-Ara*f-*(1→	H	5.16	4.23	4.10	4.23	3.74	
		C	108.62	84.28	76.47	80.76	65.80	
D	→3,5)-*α*-Ara*f-*(1→	H	5.08	4.21	4.06	3.96	3.82	
		C	106.45	78.52	80.45	83.29	65.76	
E	→4)-*α*-GalA*p-*(1→	H	5.01	3.66	4.04	4.06	4.69	
		C	98.64	67.33	69.35	81.61	70.44	174.32
F	→4)-*α*-GalA*p6*Me-(1→	H	4.85	3.67	4.04	4.02	4.74	
		C	99.39	68.70	67.71	81.68	70.44	170.35
G	→2)-*α*-Rha*p-*(1→	H	5.18	4.35	3.94	3.29	3.58	1.17
		C	98.22	75.91	68.04	72.42	69.27	15.88
H	→2,4)-*α*-Rha*p-*(1→	H	4.90	4.33	4.02	3.96	3.56	1.23
		C	98.09	75.91	73.44	76.07	70.87	16.08
I	→3)-*β*-Gal*p-*(1→	H	4.41	3.61	4.01	4.07	3.63	3.75
		C	102.59	72.12	83.24	67.71	74.24	60.40

The notations tI and tII represent specific glycosidic linkage positions in the Ara*f* residues. tI refers to the terminal (non-reducing end) position of the first Ara*f* residue. tII refers to the terminal (non-reducing end) position of the second Ara*f* residue.

**Table 3:** ^1^H and ^13^C NMR spectral data assignments of WATP-A3b structural units.

Residues	Glycosidic Linkage		1	2	3	4	5	6
A	*α*-Ara*f-*(1→^AtI^	H	5.00	4.01	3.88	4.06	3.65	
		C	106.92	83.40	75.97	80.50	60.38	
B	*α*-Ara*f-*(1→^AtII^	H	5.11	4.01	3.88	4.06	3.74	
		C	106.34	83.34	75.97	80.50	60.44	
C	→5)-*α*-Ara*f-*(1→	H	5.16	4.23	4.03	4.22	3.72	
		C	105.85	84.28	75.62	80.85	67.06	
D	→3,5)-*α*-Ara*f-*(1→	H	5.07	4.21	4.05	3.96	3.82	
		C	106.49	78.50	80.50	83.30	67.06	
E	→4)-*α*-GalA*p-*(1→	H	4.98	3.67	4.97	4.13	4.68	
		C	98.60	67.43	70.02	81.69	70.44	174.32
F	→4)-*α*-GalA*p6*Me-(1→	H	4.83	3.67	4.04	4.02	4.73	
		C	99.53	68.70	69.36	81.73	70.53	170.35
G	→2)-*α*-Rha*p-*(1→	H	5.16	4.34	3.92	3.27	3.58	1.16
		C	98.11	75.90	69.36	72.26	71.90	15.88
H	→2,4)-*α*-Rha*p-*(1→	H	4.93	4.34	4.01	3.95	3.54	1.23
		C	96.90	75.90	73.67	76.54	70.97	16.07
I	→3,6)-*β*-Gal*p-*(1→	H	4.56	3.62	3.96	4.07	3.70	3.83
		C	103.23	74.80	83.40	67.88	75.09	68.32

The notations tI and tII represent specific glycosidic linkage positions in the Ara*f* residues. tI refers to the terminal (non-reducing end) position of the first Ara*f* residue. tII refers to the terminal (non-reducing end) position of the second Ara*f* residue.

**Table 4:** Ellipticity analysis of WATP-A2b and WATP-A3b.

Pectin	[θ]_max_/deg·cm^2^·dmol^−1^
WATP-A2b	8.26
WATP-A3b	11.74

**Table 5:** Yield, molecular weight, and monosaccharide profiles of the E1–E3 fractions obtained from enzymatic hydrolysis of WATP-A2b and WATP-A3b.

Fractions	Yield ^a^ (%)	TBA Test	Molecular Weight (kDa)	Monosaccahride Composition (mol%)							
				GalA	Rha	Gal	Ara	Glc	GlcA	Man	Xyl
WATP-A2b-D-E1	50.5	−	31.1	16.6	16.8	22.6	37.3	2.1	1.8	2.8	-
WATP-A2b-D-E2	13.9	+	8.3, 5.2	28.8	17.3	11.6	15.1	20.4	5.3	1.5	-
WATP-A2b-D-E3	35.6	−	<2.0	96.6	-	0.1	0.5	1.2	0.2	1.1	0.3
WATP-A3b-D-E1	39.7	−	45.3	28.3	27.7	17.3	11.4	5.9	4.8	4.6	1.0
WATP-A3b-D-E2	17.6	+	9.4, 5.7	33.5	19.3	12.6	14.7	7.2	8.6	2.4	1.7
WATP-A3b-D-E3	42.6	−	<2.0	95.3	-	1.1	0.4	1.0	0.2	1.5	0.5

^a^ Yield based on WATP-A2b or WATP-A3b. TBA: Thiobarbituric acid.

## Data Availability

Data are contained within the article.
